# Academic Career Development of Chinese Returnees With Overseas Ph.D. Degrees: A Bioecological Development Perspective

**DOI:** 10.3389/fpsyg.2022.859240

**Published:** 2022-04-18

**Authors:** Dan Liu, Yuwei Xu, Tongtong Zhao, Siqi Che

**Affiliations:** ^1^School of English Education, Guangdong University of Foreign Studies, Guangzhou, China; ^2^Faculty of Business and Law, Coventry University, Coventry, United Kingdom; ^3^School of Education, University of Nottingham, Nottingham, United Kingdom; ^4^UNESCO International Research and Training Centre for Rural Education, Faculty of Education, Beijing Normal University, Beijing, China; ^5^School of Foreign Languages, South China University of Technology, Guangzhou, China

**Keywords:** Chinese returnees with overseas Ph.D. degrees (CROPs), academic career development, bioecological development, higher education, China

## Abstract

This study uses the bioecological model of human development to understand the academic career development of Chinese returnees with overseas Ph.D. degrees (CROPs). Focuses are placed on how CROPs engaged in this process through interactions with contexts, which lead to their differentiated and similar career development in Chinese higher education. Using a qualitative approach of semi-structured interviews with 31 CROPs, our findings reveal that CROPs’ academic career development is co-shaped by personal characteristics and multi-layered environmental contexts. The study highlights the dysfunctionality of Chinese higher education system in the context of China’s ambition to build First-class Universities and First-class Subjects (Double First-class), which constrains CROPs’ academic career development. The paper offers important implications for potential CROPs, policy, and future research studies.

## Introduction

Over the past two decades, the Chinese government has introduced a series of talent programs to attract return of its leading overseas talents, who possess Ph.D. degrees normally gained from world top universities (Li and Xue, 2021). Those programs include the “One Hundred Talents Program,” “One Thousand Talents Program” and “Yangtze River Scholars Program” (Zhang, 2019), characterized with lucrative material incentives including competitive salary packages, research funding, and other social benefits (e.g., housing, children’s education, and jobs for spouses). For example, the Thousand Talents Program launched in 2008 by the General Office of the Central Committee of the Chinese Communist Party offers 1 million RMB (143,800 USD) living allowance and a minimum of 10 million RMB (1,438,000 USD) start-up funding for setting up laboratories (Chen, 2017). It was reported that the Thousand Talent Program alone has attracted more than 8,000 talents to return by 2018 (Ren and Liu, 2019).

Despite the great effort to attract Chinese returnees with overseas Ph.D. degrees (CROPs), little attention has been put on understanding the process of re-entry experienced by them in the context of Chinese higher education (Chen, 2017). Hao et al., 2017 analyzed 143 empirical studies on Chinese returnees (including but not limited to CROPs) from 2005 to 2015 and pointed out that “what is less known and less understood are the reasons that motivate the returnees to come back, how they are doing after returning and what could be done further to attract them back and retain them” (p. 144).

While studies on reasons for Chinese individual migration and return migration are increasing, they predominantly use the push-pull theory with surveys or mixed methods of surveys and interviews (e.g., Mazzarol and Soutar, 2002; Li and Bray, 2007; Hao et al., 2017; Liu and Morgan, 2017). For example, Liu and Morgan (2017) summarized the main pull factors for students’ migration, which include high quality education, authentic English language environment, and better employment opportunities abroad. The main push factors for students’ migration include a lack of access to satisfying domestic higher education, and influence of families, friends, and agents in China. For return migration, Hao et al., 2017) classified both the push and pull factors in China into personal, professional, and societal factors. The personal push factors include limited educational opportunities for their children and lack of social connections while the personal pull factors can be close family ties at home and loneliness abroad. The professional push factors include unfavorable work culture, lack of resources, etc., and the main professional pull factor is more career opportunities in China. At societal level, culture values in China can be a push factor while the culture shock abroad and feeling of nationalism and alienation are the main pull factors. These studies highlight that both individual migration and return migration are the outcomes of rational consideration of the pull and push factors, with education, economic and career opportunities, social networks (including families and relatives), and culture values being the most important.

Studies explaining returnees’ post-return experiences are also emerging and often use theory of capitals, such as human capital, cultural capital, social capital etc. (Gill, 2010; Hao et al., 2016; Hao and Liu, 2017; Singh and Jack, 2018), transnationalism (van Meeteren et al., 2014; Gu and Schweisfurth, 2015; Setrana and Tonah, 2016; Lei and Guo, 2020; Singh, 2020), and acculturation/adjustment (Presbitero, 2016; Ai and Wang, 2017; Ai, 2019; Hoang and Ho, 2019). Our paper contributes to this research gap by employing the under-utilized theoretical framework of bioecological model of human development (Bronfenbrenner, 1979; Bronfenbrenner and Morris, 2006) to examine the process of re-entry experiences of CROPs. Focuses are not placed on the motivations for their return migration but on a comparatively less explored topic-how CROPs engaged in interactions with different environmental contexts and variables, leading to differentiated academic career development.

In the rest of this paper, we start with introducing the Chinese higher education context, in which CROPs’ career development is situated after they return to China. This is followed by an overview of current research on CROPs’ re-entry experience, where we highlight the need for a bioecological perspective to understand how CROPs experience career development. We then explain how the bioecological model of human development is adopted in this study. Guided by this theoretical framework, we elucidate our research methodology of an interpretivist approach; whereby the process of re-entry and career development is understood through the narratives of CROPs themselves. Findings and discussion follow. This paper has important implications for CROPs’ career development in the political context of China’s ambitions to establish First-class Universities and First-class Subjects in the world (Song et al., 2021). Corresponding recommendations are made for policy makers in Chinese higher education.

## Background Literature

### Chinese Higher Education: A Hierarchical Academic Structure

Chinese higher education is characterized with close supervision and control from the nation-state, as well as an authoritative, top-down, and hierarchical management system within higher education institutions (HEIs; Marginson, 2011). In those contexts, recent national higher education policies such as the Double First-class Project (to establish world-class Chinese universities and subjects) have imposed much pressure on Chinese universities competing for inclusion in this project, with substantial funding provided by the central government (Song et al., 2021). To be included in this project, Chinese universities are particularly eager for high numbers of publications in international and national journals indexed by Science Citation Index (SCI), Social Sciences Citation Index (SSCI), Chinese Science Citation Index (CSCI), and Chinese Social Sciences Citation Index (CSSCI), especially those with high impact factors (Zhang and Sivertsen, 2020; Li and Xue, 2021). Despite recent national policies has been introduced to lessen the emphasis on SCI/SSCI publications but more emphasis on the importance of publications in domestic journals, such as CSSCI (Chinese Social Sciences Citation Index), many universities in China still use the SSCI- or SCI-related metrics as an important basis (if not only) for research evaluations, for its convenience and apparent objectivity (Xu X. et al., 2021). As for the national/provincial grants-rewarding system in China, there has long been a systemic bias toward the natural sciences and technology compared to humanities and social sciences. Meanwhile, Chinese academics have to align their academic pursuits with national interest to increase their likelihood of their proposal being funded by the nation/government. The pressure is then translated by Chinese HEIs into a performance-based evaluation system that relies primarily on numbers of high-quality publications and national/provincial funding awards, which determines individual academic’s contractual employment, promotion, and overall career development (Li and Shen, 2020; Zhang and Sivertsen, 2020). It also links to other aspects of academics’ life such as their children’s access to high-quality educational settings (Li and Shen, 2020).

Meanwhile, the highly competitive HEI environment in China is worsened by a hierarchical and network-oriented culture in which those powerful have absolute authority in the allocations of resources, with priorities oftentimes given to those within their own networks. For instance, it is reported that subjective peer review process in top Chinese journals is characterized with strong back-door relationships (*guanxi*) among Chinese academics (Li, 2020). Research has suggested that CROPs are usually excluded from those networks, having spent much time abroad without the opportunities to develop relationships with big “figures” (well-established researchers in China who control allocation of competitive academic resources) in their fields (Chen, 2017; Singh, 2020; Pham, 2021). How CROPs experience and negotiate their career development in such a hierarchical system is a key theme that emerges from our findings, which is presented later in this paper.

### CROPs’ Academic Career Development

Studies on Chinese returnees’ career development prevailingly focus on their (re)adjustment and challenges. For example, Singh (2020) used a qualitative study to explore the challenges faced by Chinese graduate returnees from Australia in obtaining employment in China. Those challenges include limited working experience, a lack of *guanxi* (social networks), and unsynchronized graduation with employment months in China. Chen (2017) found that with limited time and resources, CROPs have difficulty in striking a balance between publishing articles in international and Chinese journals. Consequently, their integration into Chinese academic system is not always smooth. Li et al. (2018) explored the experiences of CROPs attracted by China’s Thousand Youth Talents Scheme (a type of talent program targeted at attracting overseas talents below 40) and found that they are confronted with high pressure, heavy workloads, bureaucratic and administrative management, and over-emphasis on quantitative evaluation of academic performance and ranking. Ai and Wang (2017) used autoethnography to narrate the first author’s experiences of integration as a returnee and highlighted the challenges of identity negotiation for academic returnees in Chinese HEIs. These studies, however, largely consider returnees as “in deficit” (Xu et al., 2020, p. 2), focusing on the influence of external factors but neglecting the interaction of the individual with environment - a process that CROPs actively engage through embodiment of personal characteristics, including *dispositions, demands*, and *resources* (Bronfenbrenner and Morris, 2006) in their career development. Therefore, we adopt Bronfenbrenner’s bioecological systems theory (Bronfenbrenner, 1979; Bronfenbrenner and Morris, 2006) to explore how CROPs manifest personal characteristics in interactions with their ecological systems, which then shape their career development.

## Bronfenbrenner’s Bioecological Framework for Crops’ Career Development

Bronfenbrenner’s (1979) ecological systems theory situates human development within a set of nested environmental contexts. There are four layered contexts including the *microsystem* (as the innermost layer consisting of activities and interactions in a person’s immediate surroundings, e.g., family, school, peers and workplace), *mesosystem* (emphasizing the interactions among those immediate settings, e.g., family-school/workplace links), *exosystem* (events in one or more environments affecting the individual’s development but without the individual’s direct participation, e.g. community, parents’ social network and workplace) and *macrosystem* (the broader cultural and national context), which jointly influence human development. For CROPs’ academic career development, the *microsystem* refers to their interaction with colleagues and line managers in the HEIs they work, as well as interaction with family members that impacts on their career decisions. The *mesosystem* involves interactions between CROPs’ work and family life, such as their children’s access to quality education provided by Chinese HEIs.^1^ The *exosystem* can include for example, academic networks that CROPs themselves do not involve, but have indirect impacts on their careers (e.g., obtaining funding sources and publishing journal articles). The *macrosystem* encompasses broader cultural, political, and economic contexts, which in this case relates to Chinese higher education system described above and other cultural beliefs and societal values that powerfully normalize CROPs’ personal experiences and development in the Chinese context. These four systems are interrelated in influencing CROPs’ career development.

Acknowledging the influences of the ecological systems above, we further embrace in our theoretical framework Bronfenbrenner’s later theoretical development of bioecological system (Bronfenbrenner and Morris, 2006). This system considers the individual as an active agent in proximal processes - the daily interactions between an individual and their ecological systems. It also recognizes the important role of personal characteristics, namely *dispositions, demands*, and *resources*, in person-environment interactions. *Dispositions* include CROPs’ career motivations and individual perceptions in different aspects of life, such as a preference for stability in work and life, which can facilitate or prevent their return and subsequent career development (Crawford et al., 2020; Xu et al., 2020). *Demands* refer to CROPs’ characteristics that elicit immediate reactions from them in their ecological systems and produce change based on these reactions (Xu et al., 2020), such as the need of establishing and maintaining academic social networks after return. *Resources* means those facilitating CROPs’ career development, such as ability, knowledge, skills, experience and personal networks, and those impeding factors where the (aforementioned) facilitators are absent (Xu et al., 2020).

Bioecological systems theory has been used to address international doctoral students’ acculturation (e.g., Elliot et al., 2016a,b; Singh and Jack, 2018; Xu et al., 2020). These studies confirm that international doctoral study experiences are co-shaped by individuals’ personal characteristics and the multi-layered bioecological system they situate in. However, research rarely uses this framework to explore international doctoral students’ (especially CROPs’) re-acculturation/re-entry experiences. A bioecological approach is therefore used in this paper to provide a comprehensive understanding of the person-environment interactivity in CROPs’ navigation of their career development after return. We address two research questions:

(1) What are the personal characteristics (*dispositions*, *demands*, and *resources*) displayed by those CROPs in their process of academic career development?
(2) How do those personal characteristics interact with the multi-layered bioecological systems in shaping CROPs’ academic career development?

## Research Methods

A qualitative, narrative approach was adopted to understand how CROPs engage in the process of re-entry and career development. Semi-structured interviews were conducted, where CROPs displayed their personal characteristics in interactions with multi-layered bioecological systems in Chinese higher education context. The interview questions centered around: “Why did you come back?,” “Can you talk about the factors positively or negatively shaping your re-entry experience and academic career development?,” “Why do you think they represent a positive or negative experience?” and ‘How have these experiences impacted on your re-entry journey and academic career development?’ Supplementary questions were asked based on what emerged in the interviews.

### Sampling and Participants

We used snowball sampling (Baltar and Brunet, 2012) to approach participants online via WeChat, a multi-purpose instant messaging and social platform widely used in China enabling us to access CROPs without geographic limitations. Specifically, the authors initially contacted four qualified subjects through their own social networks using WeChat and asked whether they would like to participate in the study. Two subjects declined because of a lack of time for the interview. The rest two agreed to participate in the study. The two participants were then asked to help to identify other qualified subjects through their social networks using WeChat. Finally, 31 CROPs from different HEIs across China volunteered to participate in the study. Participant information sheets and consent forms were distributed to them, and informed consent was obtained before the interview. The participants are diverse in demographic backgrounds (such as age, gender, marital status) and have obtained their Ph.D.s from various countries outside China (e.g., Australia, United States, Denmark, United Kingdom, New Zealand, Netherlands, France, Germany, and Japan). When the fieldwork was conducted, they were working in different cities such as Beijing, Shanghai, Guangzhou, Shenzhen, etc., as shown in Appendix Table 1. Eighteen participants were females and thirteen were male. The majority were under 35 years old, with six participants aged above 35. Participant CROPs had various funding sources to study abroad, ranging from self-funded to half and full scholarship. Most of them returned before 2018 and had lived in China for at least over 1 year when this study was conducted. It should be noted that our research was never aimed at generalizing results to the whole CROP group. Instead, we seek to explore interactions between CROPs’ personal characteristics and bioecological systems shaping their academic career development.

### Data Analysis

**FIGURE 1:**
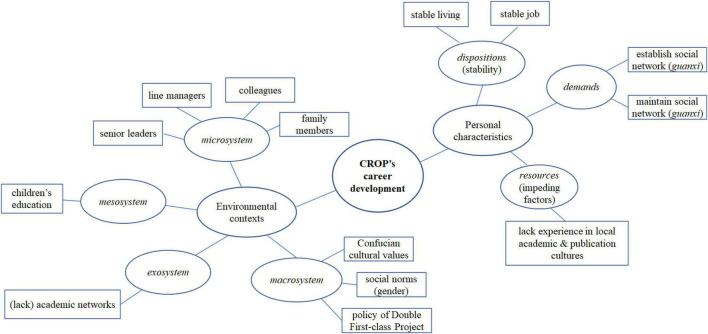
Final thematic map.

All interviews were conducted, recorded, and transcribed in Chinese mandarin by authors of this paper, who are Chinese native speakers. Thematic analysis (Clarke and Braun, 2017) was followed, mainly driven by research questions and the theory of Bronfenbrenner’s bioecological development, in which the data were coded around the personal characteristics (*dispositions*, *demands*, and *resources*) and environmental contexts (*microsystem*, *mesosystem*, *exosystem*, and *macrosystem*) influencing CROP’s career development. To be specific, firstly, the author transcribed the data and read them repeatedly to get familiar with the data. Secondly, initial codes were generated based on the data centered around the research questions. For example, one participant mentioned “Personally, I value stability most in job seeking,” which was initially coded as “stable job.” Another participant pointed out that “I want a stable place of living as I have experienced many times of moving home and do not want it anymore,” which was coded as “stable living.” These codes were then sorted into a potential theme “stability” and was later revised as “dispositions: (preferences for) stability” with reference to Bronfenbrenner’s theory. At this stage, a thematic map showing the relationship between codes and themes (and sub-themes) was produced, which was then refined into the final thematic map presented in Figure 1, showing two main categories: personal characteristics and environmental contexts. Finally, related extracts representing the main (repeated) themes that are related to the research questions are presented. Inter-researcher checks were carried out at stages of developing codes (themes) and throughout the whole process of data analysis. The first and third authors are CROPs themselves currently working in Chinese universities. The second author is a Chinese native based at a United Kingdom institution, whereas the fourth author completed her Ph.D. in China and has been working in a Chinese university afterwards. This diversity of research team with different backgrounds in careers, gender, and geographies enhanced rigor in interpreting data and in developing the codes. The authors with similar experiences to the participants allowed for better sensitivity to the cultural and contextual nuances in the bioecological system situating the participants. Other authors ensured that our own experiences do not result in overinterpretation of participants’ narratives. Our research followed ethical guidelines formulated by both China and United Kingdom, with an ethical approval gained from C University, United Kingdom (No. P104995). All data that identify participants are deleted or replaced by pseudonyms throughout this paper.

In the coming section, we present key themes within our theoretical framework, including two parts: personal characteristics embodied in CROPs’ re-entry experiences, and the four-layer ecological systems shaping CROPs’ academic career development.

## Findings

### CROPs’ Personal Characteristics in Navigating Their Career Development: Dispositions, Demands, and Resources

Participant CROPs displayed different and shared *dispositions*, *demands*, and *resources* while navigating their academic career development. The below patterns of personal characteristics are particularly prominent among the 31 CROPs.

#### Dispositions: Preferences for Stability

A desire for stability in work (and life) is a disposition shared by most CROPs (23 out of 31) regarding their career (and personal) development. For example, *Qi*, working as a postdoctoral researcher at a Double First-class university where performance pressures are at their most intense, mentioned that his personal preference for a stable job leads him to long for a permanent lectureship. However, as a freshly graduated Ph.D. with limited research or teaching experience, he found it difficult to achieve that aim and thus worked as a postdoctoral researcher as a compromise:

Personally, I value stability most in job seeking. I want to be a permanent lecturer in a university instead of being a postdoctoral researcher as I am now. It is not stable, and I will have to find another job in two years… Being a postdoctoral researcher also has little hope of being promoted to an associated professor. (*Qi, male, country of study: Denmark, field of study: natural science; funding: Chinese government funded*)

In addition to preference for a stable job, six participant CROPs also expressed desires for a stable living, by buying their own properties in the cities they work. *Juan* reported that she bought an apartment in Shenzhen (a tier 1 city where housing price is the highest in China) where she works, even though she is now in debt because of this purchase:

The housing price is too high in Shenzhen. I retuned in 2018 and bought an apartment with over 80,000 RMB [12,400 USD] a square meter and now it is rising to 100,000 RMB. We are now in great debt because of this purchase. I have no choice as I have two children and we have been renting a house over the past 7 years. I want a stable place of living as I have experienced many times of moving home and do not want it anymore. (*Juan, female, region of study: Hong Kong, field of study: social science; funding: Chinese government funded*)

*Juan*’s disposition of stability is also indicative of her interaction with the microsystem, where her career development is connected to her families. This interaction is discussed later. Different to *Juan*, *Hang* chose to work at a less prestigious university in a tier 2 city where house prices are lower, despite the same disposition of preferring stable life:

I did not choose to work in Shenzhen but went to Hangzhou. The most important thing is that I could not afford a house there [Shenzhen]…Even if I get 1,600,000 RMB through the Peacock Project [Shenzhen’s talent program], you are still not able to afford an apartment as the average housing price is nearly 100,000 RMB per square meter. (*Hang, Female; country of study: UK; field of study: Social science; funding: Chinese government funded*)

The above cases demonstrate how different CROPs actively interact with their environment. They may share dispositions but are situated in different environments, thus their different interactions with the environment result in different career choices.

#### Demands: Social Networking Establishment and Maintenance

In realizing that Chinese higher education is a system that embraces a hierarchical and network-oriented culture (see earlier in this paper), CROPs in our study (especially those early career academics) became particularly aware of the importance to establish individual connections with other academics, and especially with those in powerful positions. They actively demand for this key resource in their career development in Chinese higher education context. For example, *Qi* articulated:

I am starting to pay more attention to personal connections. I tried to get acquaintances with those big figures at international conferences and talk to them… When I submit a paper to a journal, I will consider whether the editor is someone I know. (*Qi, male, country of study: Denmark, field of study: natural science; funding: Chinese government funded*)

Like *Qi*, *Qing* also expressed that *guanxi* (social networks in Chinese term) is very important in influencing his career development. He emphasized social networking maintenance in his demand for career progression:

I came back as soon as I finished my study in Ireland, because I had my career foundation here [in China]. During my four years of studies overseas, I maintained regular contact with my previous employer and thus am well connected with them. After I came back, I went back to work for them and was promoted to a senior researcher smoothly. (Qing, *male, country of study: Ireland, field of study: social science; funding: self-funded and scholarship*)

#### Resources: A Lack of Experience With Local Academic and Publication Cultures as Impeding Resources

In our theoretical framework, *resources* mean both those facilitating individual development (such as ability, knowledge, skills, experience, and networks) and impeding factors where facilitators are absent. A total of 15 participant CROPs pointed to an absence of facilitators as impeding factors, including a lack of experience and familiarity with local academic, language use and publication cultures. Such impeding resources are evident specifically when compared with locally trained Ph.D.s who are more accustomed to those cultures. As illustrated by Chong:

Compared to locally trained Ph.D.s, we have few advantages. It’s difficult for us to publish in a Chinese journal, as we are not familiar with Chinese way of writing academic papers. It requires a long time of training to get adapted to its academic, language and publication culture. (*Chong, male, country of study: Ireland, field of study: social science; funding: self-funded*)

Similarly, *Lily* expressed her frustration about research funding applications due to a lack of familiarity with and experience in writing bids in China:

I haven’t obtained any funding since I came back, although I have tried several times. I think this might relate to my way of writing the bid. I am not familiar with the discourse in China. It seems that they want your writing bid to connect with the government document and their articulation of problem, […] quite different from that of abroad. (*Lily, female, country of study: Australia; field of study; social science; funding: Chinese government funded*)

The above findings suggest that CROPs’ career development is reported by themselves to have been shaped by some shared *dispositions*, *demands* and *resources*. However, as already shown in the dispositions, CROPs may manifest different interactions between their personal characteristics and the multi-layered ecological systems that situate them, shaping their different career development as presented below.

### CROPs’ Academic Career Development: Interactions With Multi-Layered Ecological Systems

CROPs’ interactions within different layers of ecological systems (*microsystem*, *mesosystem*, *exosystem*, and *macrosystem*) highlight the main challenges they face in navigating their academic career development.

#### Microsystem: Interactions With Senior Leaders, Line Managers, Colleagues, and Family Members

The *microsystem* where CROPs’ immediate career development takes place primarily includes their workplaces. Their interactions with senior leaders, line managers, and colleagues play an important role in shaping their re-entry experience and conceptualization of psychological well-being. Such interactions mainly exerted negative influences and pressure that CROPs found difficult to engage with. Nine CROPs consistently mentioned the pressure of publishing world leading journal articles imposed on them by their departments, as exemplified below:

I feel very pressured since I returned. As soon as I returned, the leaders in my department asked me whether I have published any paper or secured any funding. They have certain expectation toward me since I obtained my Ph.D. degree overseas. However, it is not easy to publish a paper in a short period. I become hectic at work and feel exhausted as I had other tasks, such as teaching and preparing for title evaluation. (*Wu, female, country of study: New Zealand, field of study: social science; funding: self-funded*)

An inconsiderate and exploitative line manager who manipulates (usually junior) CROPs to complete tasks for their own benefits further discouraged some CROPs’ morale and hampered their academic career development. *Na* at a Double First-class institution mentioned this emotionally:

I am not satisfied with my current work. I feel very painful, and I wanted to go back to the United Kingdom. I am now a postdoctoral researcher working in an academic research center. I must be on alert around the clock. It is quite often that we have meetings from 6.00 to 12.00 pm. We even had meetings from 10.30 to 12.00 pm. I do not have any time doing my own research project, as my time is fully occupied by my line manager’s projects that I am not good at. I really cannot bear it anymore. (*Na, female; country of study: UK; field of study: social science-cognitive psychology; funding: self-funded*)

Academic collaborations with colleagues constitute another important form of interactions in CROPs’ microsystem contexts. Five CROPs mentioned that their colleagues’ academic misconduct in authorship surprised them, as explained by *Lily* and *Liu*, who are both at a Double First-class institution in East and South China respectively:

Recently, I drafted an article. Then I asked a colleague (who asked me to come back to his university) whether he would like to give comments and join the authorship. What he did was changing the format of the paper and put his own name as the first author. I was very surprised as I never had such experience before. (*Lily, female, country of study: Australia; field of study; social science; funding: Chinese government funded*)

Once I drafted a 9,000-word report and wanted to submit to a journal. My dean of college asked me to let him have a look. I told him that I want to publish this in a journal and if he wants to use it, he should notify me beforehand. He promised he would. However, he published an article from the report on his own, which makes me feel quite hurt. If he had listed me as second author, I would have felt better, however, he was the only author and just wrote one sentence in the article saying, “This paper referred to the research report by A [me].” (*Liu, female, country of study: UK; field of study: social science; funding: partially self-funded*)

Both *Lily* and *Liu* reported disagreement with those academic misconducts (*dispositions*). However, there was no sign from their interviews that they could resist to such circumstances, possibly due to the strong hierarchical structure in Chinese academia (*exosystem)*, which is presented later.

In addition to workplace, CROPs’ microsystem of families also influences their career decisions such as returning to China. Seventeen participants cited caring responsibilities for their aging parents as an important factor leading to their return decision, as showed by Hu:

It [Returning back to China] may be partly because of my parents because I am the only child. I need to come back and take care of my parents. They are in the rural countryside, and it is difficult for them to go overseas and live with us abroad (*Hu, Male; country of study: Germany; field of study: natural science; funding: Chinese government funded*)

For four female CROPs, taking care of family members and especially children slowed their career development, as stated by *Huo*:

I have two children. I feel ashamed that my academic performances are not as good as those who returned at the same time. I had been feeling very anxious when I see others publish one paper after another while I am quite slow, as I spend a lot of time taking care of my two children. I have taken two years of maternity leave altogether, and I only had one third of the time spent on my scientific research work…Everyone has his own social attributes and family attributes, isn’t it? (*Huo, Female, country of study: France; field of study: Chemistry; funding: Chinese government funded*)

#### Mesosystem: Interactions Between CROPs’ Workplace and Family

CROPs’ career development is also found to be shaped by interactions between their workplace and family in the *mesosystem*. The interactions particularly concern children’s education, which is embedded in the priorities that CROPs enjoy in terms of their children’s accessibility to high-quality schooling provided by the universities they choose to work at. Six participants referred to this privilege as a key factor that shapes their choice of workplace. For example:

I came to Guangzhou mainly because I value the educational resources here for my child. The university I am working now is one of the top universities in Guangzhou and even in China, which has the top affiliated primary and middle schools. Studying at these schools almost ensures that my child could enter a top university in China. If I am working here, my child could attend these schools (*Yong*, male, country of study: UK; field of study: chemistry; funding: overseas scholarship).

Concerns were expressed by CROPs when access to quality education for their children is not provided by their employers, as illustrated by *Zhe*:

I am now working at a provincial research center in Guangzhou, and it does not have an affiliated primary school. We need to buy a school district house. It is over 80,000 RMB for a square meter. For us newly returned Ph.D.s, it is very difficult to buy an apartment in Guangzhou, not to mention the school district house. It is the biggest problem facing us. (*Zhe, female, country of study: Netherlands; field of study: social science-Chinese Law; funding: Chinese government funded*)

#### Exosystem: A Lack of Connections and Resources

In their *exosystem*, CROPs’ career development is indirectly limited by the highly localized academic networks that their colleagues who receive Ph.D. degrees from Chinese institutions have established. Twelve participants mentioned a lack of connections or interactions with famous scholars and big figures within and outside their working institutions, which locally trained Ph.D.s take advantage of. Consequently, CROPs’ career advancement, funding application, and paper publication are disadvantaged, as exemplified by *Si*, *Jin*, and *Pen*.

Compared to locally trained Ph.D.s, we lack social connections with academics and resources in China. We are fighting on our own, unlike those locally trained Ph.D.s who have their supervisors to support them. Their supervisors are often very famous scholars in China and have a very good source of connections in academia. Therefore, their career could develop very fast. However, we do not have such resources. It is even difficult for me to do empirical research in China because if you do not have certain connections, people will ignore you. (*Si, female; country of study; Japan; field of study: social science; funding: Chinese government funded*)

There are many projects limiting how many people could apply in each institution. Then, who will be selected by the institution is a problem. This is determined by those big figures in the university and opportunities are often given to the locally trained Ph.D.s who already had established a stable relationship with them. As a marginal figure [unimportant person without connections with those big figures], you will not have any opportunity, even if you are very excellent. (*Jin, Male, country of study: France; field of study: natural science; funding: government funded*)

It is difficult to get your paper published in a very good journal in China. You should have a very famous professor in your authorship. This is a very important stepping-stone. For some journals, if they found you are early starters in research, they will not send your article out for review. By contrast, the locally trained Ph.D.s could more easily get their paper published as they are supported by their Chinese supervisors who are the bigger figures in academia. (*Pen, male, country of study: UK; field of study: natural science; funding: self-funded*)

#### Macrosystem: A Hybrid of Cultural, Social, and Political Values and Norms

In our study, CROPs’ interactions with the above three layers of ecological systems are shaped by a hybrid of cultural, social, and pollical values and norms in the *macrosystem* of Chinese higher education and beyond. To illustrate, the Confucian values of family and filial piety (a central Confucian virtue referring to the attitudes of obedience, devotion, and care toward one’s parents and elder family members) acted as a strong factor pushing CROPs to return, as showed in the examples in the *microsystem* above. Those values also intersect with China’s previous political context of one-child policy (Xu and Woodyer, 2020), whereby the generation of CROPs in their 30s, being the major group of our participants, are mainly the only child who is expected to fulfill caring responsibilities for their aging parents in Chinese society. Common societal expectations of stability and quality education for children significantly influence CROPs’ choices of working institutions and locations. Additionally, the socially constructed gender roles in Chinese culture that females are primary carers of family and children (Liu and Morgan, 2016) particularly constrain female CROPs’ career ambitions and development.

Further, Chinese government’s Double First-class Project to establish world-class universities and subjects have resulted in a highly competitive and stressful working environment that CROPs interact with on an everyday basis, particularly in areas like journal publications and successful funding applications. In this political context, the cultures of hierarchical academic relationships and localized research networks (*guanxi*) in Chinese higher education are also part of the *macrosystem* that marginalizes CROPs and disadvantages their academic career development.

## Discussion

Using Bronfenbrenner’s (1979), Bronfenbrenner and Morris (2006) bioecological approach, this paper presents participant CROPs’ personal characteristics (*dispositions*, *demands*, and *resources)* and interactions with multi-layered ecological systems in the process of academic career development. Individual CROPs’ *dispositions* that prefer a stable job and living place shape their career choice of locations. A preference for stability in workplace reflects the fierce competition in enterprises and the mainstream societal values in China. Their *demand* for *guanxi* and network maintenance necessary for career development leads to some CROPs actively building and maintaining such networks in their workplace. These developmental dispositions and demands are suggestive of CROPs’ agency, efficacy, and initiative in navigating their own academic career development. However, not all CROPs could have their demands satisfied due to lack of bioecological *resources*. Their lack of recent experience and familiarity with local academic and publication cultures impeded some CROPs’ career development. Although our study reveals certain agency from the participant CROPs to address those limitations, it is to various extent constrained by the multi-layered ecological systems at micro, meso, exo, and macro levels.

The interactions with families in the *microsystem* influence CROPs’ career choices of returning to China. In the workplace, CROPs’ interactions with senior leaders, line managers, and colleagues are reported to have imposed negative influences on their everyday work, embodied as academic pressure and mental illness. For CROPs at early career stages, their interactions with exploitative line managers influenced their conceptualization of well-being and put them under great pressure. They felt much stressed and had to work overtime. Other CROPs were frustrated by their colleagues’ academic misconduct such as plagiarism and mis-claim in authorship, which is in sharp contrast to their previous experiences abroad, as echoed in Cao (2008). Those influences at micro levels are shaped by the *macrosystem*, particularly the wider hectic Chinese academic culture characterized with “rigid, quantified evaluation criteria” focusing on immediate publications and obtaining research grants (Wang et al., 2016, p. 264; Huang, 2020). CROPs’ interactions in the *microsystem* of workplace also reflect the recent changes in the *macrosystem* of Chinese higher education, with a political drive to establish world-class universities and subjects pushed by marketization and globalization in international higher education (Song et al., 2021). The dynamic interactions and connections between the different layers of contextual forces correspond with Bronfenbrenner’s (1977) argument that different layers of context are interrelated.

Such interrelations are also evident at meso and exo levels of CROPs’ ecological systems. In the *mesosystem* comprising interactions between CROPs’ workplace and family, CROPs with children oftentimes premise their choices of working universities on whether the universities provide access to quality education in university-affiliated kindergartens, primary and secondary schools. It reflects the education inequality in China because of uneven regional economic development where an equal access to quality education is denied. The connections and interactions that CROPs’ colleagues (mainly those locally trained Ph.D.s) have with powerful leaders and famous scholars in Chinese academia constitute as an *exosystem*. It facilitates non-CROP academics’ career development as the “Big Figures” (*Da Lao* 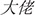 in Chinese) control allocation of competitive academic resources (Wang et al., 2016, p. 262), and indirectly limits CROPs’ opportunities in achieving career advancement, paper publication and project application due to a lack of such connections. This is echoed by Pham (2021) that social capital (*guanxi* networks in Chinese) plays an important role in returnees’ career promotion and development. As also pointed out by Li and Xue (2021), CROPs are “abandoned orphans” as they are marginalized in their local academic networks and communities.

Finally, all above-mentioned systems and interactions within are shaped by the *macrosystem* constituting Chinese cultural values and norms imposed on and embodied in CROPs’ career development. Confusion view toward families and especially the responsibility of taking care of aging parents (filial piety) exemplified as an important social belief in the *macrosystem*, prominently motivating CROPs to return and work in China. Traditionally gendered roles that men work outside home while women shoulder major domestic responsibilities and take care of children (Liu and Morgan, 2020) are another reflection of Chinese cultural beliefs in the *macrosystem* - which add worries for female CROPs and impede their career progress in Chinese higher education (Tang and Horta, 2021). The quote of “everyone has his[/her] own social attributes and family attributes, isn’t it” by one of the CROPs in this study is a typical example reflecting Chinese traditional Confucian views of gender and families. The influence of traditional views of gender and families on migrants and returnees are also noted by Baykara-Krumme and Platt (2018) and King and Lulle (2022).

## Conclusion, Implications, and Directions for Future Research

This paper has explored how CROPs’ personal characteristics and multi-layered bioecological systems interact to jointly shape CROPs’ academic career development back in China. We find that the interactions tend to act negatively for CROPs, aligning with the argument of Merçon-Vargas et al. (2020) that proximal processes in individuals’ bioecological systems do not always act positively on individual developmental outcomes. For most participants in our study, the negative influences exerted by the multiple socio-ecological systems in Chinese higher education and beyond are highly normative, which constrained individual CROP’s agency in interacting with bioecological systems, and further their ability to influence the systems. Our paper provides support for the under-utilized theoretical framework of bioecological model of human development (Bronfenbrenner, 1979; Bronfenbrenner and Morris, 2006) in examining the process of re-entry experiences of CROPs, which highlights the interplay of macro, meso and exo-level factors in shaping the micro-level experiences of CROPs’ career development. We conclude this article with implications for policy and the directions for future research.

The findings of this study have several policy implications. First, it is important to support individual CROPs in their development of agency, so that they are aware of and prepared for possible interactions with their immediate *microsystems*. Talent programs by Chinese governments to attract CROPs could use findings from this study to develop mentorship and career support schemes that empower CROPs. In so doing, CROPs’ different dispositions and demands are enabled. Second, from the perspective of *mesosystem*, a fair and accessible education system for all children in China is needed, which may downplay the influence of interactions between family and workplace on CROPs’ career choices. Obviously, this implication itself is a big issue that China needs to address in its educational policies and is beyond the scope of this study. Third, from the perspective of *exosystem*, on one hand, Chinese universities should consider assisting the inclusion of CROPs in local academic networks and communities, for example, through organizing different seminars and research workshops that truly bridges the CROPs and locally trained scholars. This is also beneficial for realizing the double First-class dream when both parties’ combined strengths are utilized. On the other, it is urgent to establish a fair and competitive academic structure rather than focusing on authority and *guanxi*. For example, it is useful to widely apply the double-blind peer review system in paper publication and research grant review processes. Fourth, from the perspective of *macrosystem*, state governments are suggested to launch funding policies targeting at supporting junior CROPs, like subsidized housing (or transitional apartments) and start-up research fund, especially in big cities where housing prices are incredibly high. Policies that address the disadvantages of women academics are extremely recommended, in aspects such as flexible working and academic evaluation standards for those with childcare responsibilities.

The paper has its limitations. Firstly, participants’ cities of origin were not investigated, but which might have an influence on their career development. Future studies are recommended to consider this and explore whether this makes a difference to the participants’ career development. Secondly, as an exploratory study with interviews only, our paper is limited by its generalizability and future studies exploring CROPs’ re-entry experiences and career development at larger scales are recommended.

## Data Availability Statement

The raw data supporting the conclusions of this article will be made available by the authors, without undue reservation, upon request to the corresponding author.

## Ethics Statement

The studies involving human participants were reviewed and approved by Guangdong University of Foreign Studies. The patients/participants provided their written informed consent to participate in this study.

## Author Contributions

DL is mainly in charge of research methodology, data analysis, and drafting of the manuscript. YX is responsible for revision of the manuscript. TZ and SC are mainly responsible for data collection and revision of the manuscript. All authors contributed to the article and approved the submitted version.

## Conflict of Interest

The authors declare that the research was conducted in the absence of any commercial or financial relationships that could be construed as a potential conflict of interest.

## Publisher’s Note

All claims expressed in this article are solely those of the authors and do not necessarily represent those of their affiliated organizations, or those of the publisher, the editors and the reviewers. Any product that may be evaluated in this article, or claim that may be made by its manufacturer, is not guaranteed or endorsed by the publisher.

## Footnotes

1 Chinese HEIs oftentimes provide their employees with access to their affiliated kindergartens, elementary and secondary schools.

## Appendix

**TABLE A1:** Participant CROPs’ demographic backgrounds.

Item	NO.
Gender	
Male	13
Female	18
Age	
30 and under	7
31–35	18
36–40	5
41–50	1
Above 50	0
Marital status	
Married	19
Single	12
Current working city	
Beijing	2
Shanghai	1
Guangzhou	14
Shenzhen	5
Other	9
Subject area	
Humanities and social sciences	16
Natural science	10
Other	5
Funding source for studying abroad	
Full scholarship	22
Half self-funding and half scholarship	4
Self-funding	5
Title	
Associate professor	11
No title	6
Other	14
Year of going abroad	
Between 2001 and 2010	4
2011 and after	27
Length of studying abroad	
2–3 years	4
4–5 years	23
5 years and above	4
Country of studying abroad	
Australia	5
United States	3
Denmark	2
United Kingdom	11
New Zealand	1
Netherlands	2
France	2
Germany	2
Japan	3

## References

[B1] Ai B. (2019). Pains and gains of working in Chinese universities: an academic returnee’s journey. *High. Educ. Res. Dev.* 38 661–673. 10.1080/07294360.2019.1590320

[B2] Ai B. Wang L. (2017). Homeland integration: an academic returnee’s experiences in Chinese universities. *Int. J. Qual. Methods* 16:1609406917696741.

[B3] Baltar F. Brunet I. (2012). Social research 2.0: virtual snowball sampling method using facebook. *Internet Res.* 22 57–74. 10.1108/10662241211199960

[B4] Baykara-Krumme H. Platt L. (2018). Life satisfaction of migrants, stayers and returnees: reaping the fruits of migration in old age? *Ageing Soc.* 38 721–745. 10.1017/s0144686x16001227

[B5] Bronfenbrenner U. (1979). *The Ecology of Human Development: Experiments by Nature and Design.* Cambridge, MA: Harvard University Press.

[B6] Bronfenbrenner U. Morris P. A. (2006). “The bioecological model of human development,” in *Handbook of Child Psychology: Theoretical Models of Human Development*, Vol. Ed eds Damon W. Lerner R. M. (New York, NY: Wiley). 793–828.

[B7] Cao C. (2008). China’s brain drain at the high end. *Asian Popul. Stud.* 4 331–345. 10.1080/17441730802496532

[B8] Chen Q. (2017). *Globalization and Transnational Academic Mobility: The Experiences of Chinese Academic Returnees.* Berlin: Springer.

[B9] Clarke V. Braun V. (2017). Thematic analysis: striving to meet the trustworthiness criteria. *J. Posit. Psychol.* 12 297–298. 10.1080/17439760.2016.1262613

[B10] Crawford B. F. Snyder K. E. Adelson J. L. (2020). Exploring obstacles faced by gifted minority students through Bronfenbrenner’s bioecological systems theory. *High. Ability Stud.* 31 43–74. 10.1080/13598139.2019.1568231

[B11] Elliot D. L. Baumfield V. Reid K. Makara K. A. (2016a). Hidden treasure: successful international doctoral students who found and harnessed the hidden curriculum. *Oxford Rev. Educ.* 42 733–748. 10.1080/03054985.2016.1229664

[B12] Elliot D. L. Reid K. Baumfield V. (2016b). Beyond the amusement, puzzlement and challenges: an enquiry into international students’ academic acculturation. *Stud. High. Educ.* 41 2198–2217. 10.1080/03075079.2015.1029903

[B13] Gill S. (2010). The homcoming: an investigation into the effect that studying overseas had on Chinese postgraduates’ life and work on their return to China. *Compare* 40 359–376. 10.1080/03057920903464555

[B14] Gu Q. Schweisfurth M. (2015). Transnational connections, competences and identities: experiences of Chinese international students after their return “home”. *Br. Educ. Res. J.* 41 947–970. 10.1002/berj.3175

[B15] Hao J. Liu Q. (2017). The impact of Australian accounting education on repatriates’ career development. *Aust. Account. Rev.* 27 52–60. 10.1111/auar.12141

[B16] Hao J. Wen W. Welch A. (2016). When sojourners return: employment opportunities and challenges facing high-skilled Chinese returnees. *Asian Pacific Migr. J.* 25 22–40. 10.1177/0117196815621806

[B17] Hao X. Yan K. Guo S. B. Wang M. L. (2017). Chinese returnees’ motivation, post-return status and impact of return: a systematic review. *Asian Pacific Migr. J.* 26 143–157. 10.1177/0117196817690294

[B18] Hoang H. T. Ho N. T. T. (2019). Antecedents of work readjustment of professional returnees: evidence from Vietnam. *Asia Pacific J. Bus. Adm.* 12 58–72. 10.1108/APJBA-05-2019-0118

[B19] Huang Y. (2020). Doctoral writing for publication. *High. Educ. Res. Dev.* 40 1–14. 10.1080/07294360.2020.1789073

[B20] King R. Lulle A. (2022). “Gendering return migration,” in *Handbook of Return Migration* (Cheltenham: Edward Elgar Publishing), 10.4337/9781839100055.00012

[B21] Lei L. Guo S. (2020). Conceptualizing virtual transnational diaspora: Returning to the ‘return’of Chinese transnational academics. *Asian Pacific Migr. J.* 29 227–253. 10.1177/0117196820935995

[B22] Li B. Shen Y. (2020). Publication or pregnancy? Employment contracts and childbearing of women academics in China. *Stud. High. Educ.* 1–13. *v, 10.1080/03075079.2020.1817888

[B23] Li J. Xue E. Y. (2021). Returnee faculty responses to internationalizing “academic ecology” for creating world-class universities in China’ elite universities. *High. Educ.* 81 1063–1078. 10.1007/s10734-020-00599-y

[B24] Li M. Bray M. (2007). Cross-border flows of students for higher education: push-pull factors and motivations of mainland Chinese students in Hong Kong and Macau. *High. Educ.* 53 791–818. 10.1007/s10734-005-5423-3

[B25] Li M. Yang R. Wu J. (2018). Translating transnational capital into professional development: a study of China’s thousand youth talents scheme scholars. *Asia Pacific Educ. Rev.* 19 229–239. 10.1007/s12564-018-9533-x

[B26] Li S. Q. (2020). *The End of Publish or Perish? China’s New Policy on Research Evaluation.* Available online at: https://www.mpiwg-berlin.mpg.de/observations/1/end-publish-or-perish-chinas-new-policy-research-evaluation on (accessed June 15, 2021)

[B27] Liu D. Morgan W. J. (2016). Students’ decision-making about postgraduate education at G university in China: The main factors and the role of family and of teachers. *Asia Pacific Educ. Res.* 25 325–335. 10.1007/s40299-015-0265-y

[B28] Liu D. Morgan W. J. (2017). “Chinese students overseas: choice of destination,” in *Handbook of Education in China*, eds Morgan W. J. Gu Q. Li F. L. (Cheltenham: Edward Elgar Publishing). 442–467. 10.4337/9781783470662.00031

[B29] Liu D. Morgan W. J. (2020). Why do students enrol for postgraduate education in China? The influence of gender and of family habitus. *Gender Educ.* 32 177–193. 10.1080/09540253.2018.1447092

[B30] Marginson S. (2011). Higher education in east asia and singapore: rise of the confucian model. *High. Educ.* 61 587–611. 10.1007/s10734-010-9384-9

[B31] Mazzarol T. Soutar G. N. (2002). “Push-pull” factors influencing international student destination choice. *Int. J. Educ. Manage.* 16 82–90. 10.1108/09513540210418403

[B32] Merçon-Vargas E. A. Lima R. F. F. Rosa E. M. Tudge J. (2020). Processing proximal processes: what bronfenbrenner meant, what he didn’t mean, and what he should have meant. *J. Fam. Theory Rev.* 12 321–334. 10.1111/jftr.12373

[B33] Pham T. (2021). Reconceptualising employability of returnees: what really matters and strategic navigating approaches. *High. Educ.* 81 1329–1345. 10.1007/s10734-020-00614-2

[B34] Presbitero A. (2016). Culture shock and reverse culture shock: the moderating role of cultural intelligence in international students’ adaptation. *Int. J. Intercult. Relat.* 53 28–38. 10.1016/j.ijintrel.2016.05.004

[B35] Ren N. Liu H. (2019). Domesticating ‘transnational cultural capital’: the Chinese state and diasporic technopreneur returnees. *J. Ethn. Migr. Stud.* 45 2308–2327. 10.1080/1369183X.2018.1534583

[B36] Setrana M. B. Tonah S. (2016). Do transnational links matter after return? Labour market participation among ghanaian return migrants. *J. Dev. Stud.* 52 549–560. 10.1080/00220388.2015.1126255

[B37] Singh J. K. N. (2020). Challenges in obtaining employment in China: lived experiences of Australian Chinese graduates. *Aust. J. Career Dev.* 29 153–163. 10.1177/1038416220947085

[B38] Singh J. K. N. Jack G. (2018). The benefits of overseas study for international postgraduate students in Malaysia. *High. Educ.* 75 607–624. 10.1007/s10734-017-0159-4

[B39] Song J. Chu Z. Xu Y. (2021). Policy decoupling in strategic response to the double world-class project: evidence from elite universities in China. *High. Educ.* 82 255–272. 10.1007/s10734-020-00642-y

[B40] Tang L. Horta H. (2021). Women academics in Chinese universities: a historical perspective. *High. Educ.* 82 865–895. 10.1007/s10734-020-00669-1

[B41] van Meeteren M. Engbersen G. Snel E. Faber M. (2014). Understanding different post-return experiences. *Comp. Migr. Stud.* 2 335–360. 10.5117/CMS2014.3.MEET

[B42] Wang Q. Tang L. Li H. (2016). “Experiences of returned chinese migrants in higher education examined through a case study,” in *Rethinking International Skilled Migration*, eds van Riemsdijk M. Wang Q. F. (Abingdon-on-Thames: Routledge), 249–267.

[B43] Xu X. Oancea A. Rose H. (2021). The impacts of incentives for international publications on research cultures in Chinese humanities and social sciences. *Minerva* 59 469–492. 10.1007/s11024-021-09441-w

[B44] Xu X. Sit H. Chen S. (2020). International education through a bioecological development lens–a case study of Chinese doctoral students in Australia. *High. Educ. Res. Dev.* 40 1–16. 10.1080/07294360.2020.1811646

[B45] Xu Y. Woodyer T. (2020). “One child policy, China,” in *The SAGE Encyclopedia of Children and Childhood Studies*, ed. Cook D. T. (Thousand Oaks, CA: SAGE Publications Inc.). 1156–1157. 10.4135/9781529714388.n426

[B46] Zhang L. Sivertsen G. (2020). The new research assessment reform in China and its implementation. *Sch. Assess. Rep.* 2:3. 10.29024/sar.15 15154348

[B47] Zhang Y. C. (2019). Making the transnational move: deliberation, negotiation, and disjunctures among overseas Chinese returnees in China. *J. Ethn. Migr. Stud.* 45 455–471. 10.1080/1369183X.2018.1459182

